# CIRCADIAN PATTERNS OF FREE-WHEEL RUNNING IN YOUNG AND OLD MICE

**DOI:** 10.1093/geroni/igz038.3322

**Published:** 2019-11-08

**Authors:** Carly Hibbs, Musharraf Yusifova, Benjamin McNair, Danielle Bruns, Emily Schmitt

**Affiliations:** University of Wyoming, Laramie, Wyoming, United States

## Abstract

The mammalian circadian clock operates on a 24-hour cycle and regulates physiological, endocrine, and metabolic responses to changes in the environment. Aging disrupts this circadian process, increasing risk for development of age-associated diseases. Free-wheel running is not only an indicator of circadian rhythm, but also a strong predictor of survival from age-related diseases (i.e. cardiovascular disease). Thus, understanding the impact of age on free-wheel running can lead to a better understanding of disease progression. We analyzed free wheel running in both male and female C57BL/6J mice at young (3-6 months) and old (18-21 months) ages exposed to standard 12h light/dark cycle. Running wheel data was recorded hourly for 10 days. As expected, young female mice ran more than male mice, and old mice ran less than young mice. Regulation of wheel running demonstrated that older mice of both sexes had a delayed start time in activity patterns. Young mice began running immediately at lights off (signaling the start of their active period) and ran consistently throughout the dark phase with peak activity in the first 2 hours. In contrast, older mice had a delayed response to light with peak activity not occurring until hours 4-6 of the dark cycle and nightly activity ending 2 hours before lights on. Ongoing work will assess the central (brain) and peripheral (muscle, cardiac) regulation of free-wheel running in aging. Together, we demonstrate the importance of studying molecular mechanisms underlying circadian misalignment in older individuals to identify ways to combat age-associated disease with circadian misalignment.

